# Generation of a DSF-Guided Refolded Bacterially Expressed Hemagglutinin Ectodomain of Influenza Virus A/Puerto Rico/8/1934 H1N1 as a Model for Influenza Vaccine Antigens

**DOI:** 10.3390/vaccines11101520

**Published:** 2023-09-24

**Authors:** Vlad-Constantin Tofan, Andreea-Laura Ermeneanu, Iuliana Caraș, Alina Lenghel, Irina-Elena Ionescu, Cătălin Țucureanu, Claudiu Gal, Crina-Georgeta Stăvaru, Adrian Onu

**Affiliations:** 1 “Cantacuzino” Institute, 050096 Bucharest, Romaniacaras.iuliana@cantacuzino.ro (I.C.); ionescu.irina@cantacuzino.ro (I.-E.I.); tucureanu.catalin@cantacuzino.ro (C.Ț.); stavaru.crina@cantacuzino.ro (C.-G.S.); onu.adrian@cantacuzino.ro (A.O.); 2 Faculty of Pharmacy, Titu Maiorescu University, 040317 Bucharest, Romania

**Keywords:** influenza, hemagglutinin, recombinant protein expression, differential scanning fluorimetry, vaccine

## Abstract

Influenza virus infections represent an ongoing public health threat as well as an economic burden. Although seasonal influenza vaccines have been available for some decades, efforts are being made to generate new efficient, flexible, and cost-effective technologies to be transferred into production. Our work describes the development of a model influenza hemagglutinin antigen that is capable of inducing protection against viral challenge in mice. High amounts of the H1 hemagglutinin ectodomain, HA_18–528_, were expressed in a bacterial system as insoluble inclusion bodies. Solubilization was followed by a thorough differential scanning fluorimetry (DSF)-guided optimization of refolding, which allows for fast and reliable screening of several refolding conditions, yielding tens of milligrams/L of folded protein. Structural and functional analysis revealed native-like folding as well as the presence of a mix of monomers and oligomers in solution. Mice immunized with HA_18–528_ were protected when exposed to influenza A virus as opposed to mice that received full-length denatured protein. Sera of mice immunized with HA_18–528_ showed both high titers of antigen-specific IgG1 and IgG2a isotypes as well as viral neutralization activity. These results prove the feasibility of the recombinant bacterial expression system coupled with DSF-guided refolding in providing influenza hemagglutinin for vaccine development.

## 1. Introduction

Influenza virus infections represent an ongoing major public health issue inflicting high numbers of hospitalizations and casualties, with the most vulnerable groups consisting of children, elders, and immunocompromised individuals [1]. As of December 2017, influenza-related respiratory deaths per year were estimated to be 290,000–650,000 worldwide [2], while preliminary in-season estimates for the most recent flu season in the United States reached 27–54 million illnesses and 19,000–58,000 deaths [3]. High infection rate peaks occur periodically, usually once per year, in each hemisphere due to continuous antigenic drift that hinders long-lasting immunity. Occasionally, highly pathogenic influenza strains may gain widespread propagation, causing a pandemic emergency [4,5,6]. The most recent flu pandemic was caused by influenza A H1N1 in 2009 and resulted in approximately 290,000 related deaths [7], yet deadlier events have occurred, the most representative in the past century being the 1918 Spanish Flu, which caused approximately 50 million deaths while also exhibiting a unique mortality age pattern [8,9].

Despite the availability of flu-specific treatments, their effectiveness is limited, rendering prevention through vaccination the most adequate tool in battling symptoms onset and infection spread [10,11]. The first inactivated influenza vaccines were developed during the 1940s, after the isolation and identification of the influenza virus in 1933. Nowadays, the manufacturing of flu vaccines consists of roughly the same core technology, meaning the use of egg-based or cell culture-based virus propagation and chemical inactivation with or without fragmentation of viral particles [12,13]. Although robust, this technology is susceptible to vulnerabilities regarding reliance on egg availability, the acquisition of genome mutations during virus propagation, and slow reaction times to viral strain updates [14,15]. Moreover, there are concerns regarding the high degree of reactogenicity, low immunogenicity, bioburden, and allergic reactions [16].

An alternative to viral propagation for vaccine manufacturing is the use of recombinant DNA technologies for producing pure viral antigenic protein subunits, thus avoiding dealing with the infectious agent directly. A quadrivalent formulation of influenza hemagglutinins produced in Sf9 insect cells is already approved for human use in individuals older than 18 years [17].

Expression of recombinant proteins in insect cells and mammalian cells usually generates high yields, yet, in terms of the highest conceivable amounts (tens of milligrams/L), the most lucrative protein expression hosts are *E. coli*-based strains. Still, as tempting as it is, protein expression in bacterial hosts, especially when dealing with proteins for human use, has been met with reluctancy due to poor folding, a lack of post-translational modifications, and potential contamination with bacterial components [18,19,20]. Numerous studies have tackled these issues by developing multiple protein folding strategies and tools to evaluate and recognize native-like protein conformations [21], while there is also evidence that some post-translational modifications rather hinder a more efficient immune response [22,23].

This study describes the production of an influenza A virus hemagglutinin ectodomain variant by means of bacterial expression, followed by a differential scanning fluorimetry-based protein refolding and stabilization screen and affinity purification. The protein solution consisted primarily of protein oligomers, which exhibited biological activity in vitro and induced protection against a viral challenge in BALB/c mice. Immunization with refolded HA_18–528_ was shown to induce a balanced antibody response and generate neutralization activity against the H1N1 influenza virus.

## 2. Materials and Methods

### 2.1. Chemicals and Materials

**Figure 1:**
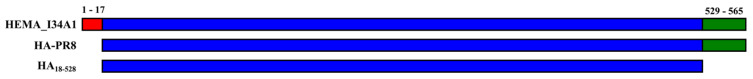
The hemagglutinin extracellular domain, HA_18–528_, was derived from full-length hemagglutinin, HA-PR8, of influenza A/Puerto Rico/8/1934 H1N1 by the deletion of amino acids from 529 to 565, corresponding with the transmembrane and cytoplasmic domains. HEMA_I34A1 corresponds to the full amino acid sequence of hemagglutinin in influenza A/Puerto Rico/8/1934 H1N1.

Reagents were generally acquired from Sigma-Aldrich and ThermoFischer Scientific. A previously assembled pET-24a(+) plasmid containing the coding sequence for full-length influenza hemagglutinin (without signal peptide) [A/Puerto Rico/8/1934 H1N1] with a hexahistidine tag was used as a template for sequence modifications. The encoded protein, hereinafter referred to as HA-PR8 (Figure 1 and Supplementary Figure S1), was produced by bacterial expression as inclusion bodies that were later solubilized in Laemmli sample buffer and purified by preparative SDS-PAGE. Analytical SDS-PAGE and DSF analysis of purified HA-PR8 are shown in Supplementary Figures S2 and S3.

### 2.2. Construction of Expression Vector

pET24a-(+) vector harboring the gene encoding the influenza virus A/Puerto Rico/8/1934 H1N1 hemagglutinin extracellular domain (HA_18–528_) (Figure 1, Table 1 and Supplementary Figure S1) was generated by modifying the pET-24a(+) plasmid containing the gene for HA-PR8. We employed a heterostagger PCR [24] coupled with a ligation-independent cloning (LIC) protocol in order to excise the sequence corresponding to the transmembrane and the cytoplasmic domain and to join the free ends, restoring the circular form. Two separate reactions were performed using two different primer pairs. The first reaction was performed using forward primer 5′-CACCACCACCACCACCACTGAGAT-3′ and reverse primer 5′-CTGATAGATCCCCATTGATTCCAATTTCACTCC-3′, while the second reaction used forward primer 5′-CACCACTGAGATCCGGCTGC-3′ and reverse primer 5′-GTGGTGGTGGTGCTGATAGATCCCC-3′. The PCR mixture (25 μL) contained 1×xPhusion HF Buffer, 1U Phusion Hot Start II DNA Polymerase, 31.25 ng of template DNA, 0.5 nM of each primer, and 200 μM of each dNTP. The amplification parameters consisted of an initial denaturation at 98 °C for 2 min, followed by 40 cycles of denaturation at 98 °C for 10 s, annealing at 64 °C (first reaction) or 62 °C (second reaction) for 20 s, extension at 72 °C for 3 min, and a final extension at 72 °C for 7 min. The PCR-amplified products were run on a 0.8% agarose gel and visualized by ethidium bromide staining, excised from the gel, and purified using the “Zymoclean Gel DNA Recovery Kit”. The two purified PCR products were mixed in an equimolar ratio to a final total quantity of 100 ng. Polymerase buffer was added, and the mix was subjected to a hybridization reaction that consisted of denaturation at 95 °C for 3 min, followed by 4 cycles of 65 °C for 2 min and 25 °C for 15 min. *E. coli* TOP10 cells were transformed with a hybridized mix and plated on LB agar plates containing 30 μg/mL kanamycin. The resulting colonies were PCR-screened with T7 primers, and the plasmid was purified.

### 2.3. Recombinant Protein Expression in E. coli

pET24a-(+)-HA_18–528_ was transformed into *E. coli* Bl21(DE3) cells by the heat shock method [25]. A single colony was picked from a freshly transformed plate, inoculated in 2 mL of LB broth containing kanamycin, and grown overnight at 37 °C with shaking. The pre-culture was transferred into 500 mL of 2YT growth medium and incubated under the same conditions. Overexpression was induced by adding 1 mM IPTG when the culture reached an optical density of 0.8–1.1. After 3 h, cells were harvested by centrifugation at 13,000× *g* for 15 min at 4 °C.

### 2.4. Inclusion Body Purification and Solubilization

Cells were treated with a cold osmotic shock to remove the periplasmic fraction [26]. The resulting pellet was resuspended in lysis buffer (0.1% Triton X-100, 50 mM NaCl, 25 mM Tris-HCl, pH 8) and lysed on ice by ultrasonication for 20 min (ν = 0.6 s^−1^, A = 100%). The lysate was centrifuged for 15 min at 10,000× *g* at 4 °C. The insoluble fraction was further washed eight times with lysis buffer (except for final washing, when Triton X-100 was omitted) and ultrasonicated for 5 min, followed by centrifugation. Washed inclusion bodies were resuspended overnight at 4 °C in 20 mL of solubilization buffer (4 M guanidinium hydrochloride, 25 mM Tris-HCl, pH 8). Mild ultrasonication on ice was used to disperse the remaining aggregates. The solution was centrifuged, and the supernatant with solubilized protein was collected, filtered through a 0.22 μm membrane, and brought to a final concentration of 2 mg/mL.

### 2.5. Differential Scanning Fluorimetry Guided Refolding

Differential scanning fluorimetry (DSF) was used to screen for optimal refolding conditions [27]. Screening was performed by a 1:20 rapid dilution of solubilized protein solution in 1 mL of various refolding buffers (Supplementary Table S1) and was incubated at 4 °C. At selected timepoints (1 h, 3 h, 18 h, 24 h, 48 h, 72 h, and 168 h), samples of each refolding mixture were mixed with SYPRO Orange Protein Gel Stain 5000× (ThermoFisher Scientific) to a final concentration of 5×. The mix was heated from 25 °C to 95 °C with a heating rate of 2 °C/min, and fluorescence was recorded at 1 °C using a BioRad C1000 Touch/CFX96 thermal cycler in FRET mode. T_m_ (melting temperature), calculated as the maximum of the fluorescence first derivative, and the amplitude of the change in fluorescence were the parameters that were used to rank refolding buffer candidates. The best refolding buffer candidates were selected as starting points for a second round of screening in which the influence of additives (NaCl, PEG 3350, oxidized/reduced glutathione ratio) was evaluated (Supplementary Table S2). A solution of protein refolded in optimal conditions was filtered through a 0.22 μm membrane, concentrated to 2 mg/mL by ultrafiltration (NMWL: 30 kDa), and once again diluted 1:20 in dialysis buffers (Supplementary Table S3) that differ by pH and NaCl concentrations, incubated at 4 °C, and analyzed by DSF at different timepoints.

### 2.6. Protein Purification and Characterization

When scaling up, refolded protein was dialyzed against optimal dialysis buffer without prior concentration. Instead, after dialysis, concentration and purification were carried out simultaneously on a HisTrap FF column using an ÄKTA start protein purification system (Cytiva, Little Chalfont, UK) and gradient elution with imidazole. Afterwards, the eluate was cleared of imidazole on a HiTrap Desalting column, filtered through a 0.22 μm membrane under sterile conditions, and stored at 4 °C. Protein concentration was quantified with the Qubit™ Protein Assay (ThermoFisher Scientific, Waltham, MA, USA). SDS-PAGE was used to assess the presence and purity of HA_18–528_ throughout the expression, processing, and purification steps. Blue Native (BN)-PAGE was performed as described by Wittig et al. [28], and particle size distribution analysis was carried out on a Zetasizer Nano ZS (Malvern Panalytical, Malvern, UK) with backscatter detection. Analytical Field Flow Fractionation (FFF) was performed with an Eclipse Long Channel equipped with a 400 μm spacer and a 10 kDa regenerated cellulose membrane under the control of a Wyatt Eclipse system. System flow was controlled by Agilent 1260 Infinity II pumps, and UV detection was carried out on the Agilent 1260 Infinity II VWD. A Wyatt DAWN 8 multiple-angle light scattering (MALS) detector was used inline to collect the scattering signal. Data analysis was performed in ASTRA 8 using 280 nm UV as a concentration source, and scattering data was fit to an order 1 Zimm model to calculate the molecular weight and radius of gyration for the eluting particles. Mass analysis was performed on an autoflex speed MALDI TOF-TOF mass spectrometer (Bruker, Billerica, MA, USA) using samples that were previously treated with trypsin (Promega, Madison, WI, USA) and separated using a Dionex Ultimate 3000 RSLCnano system on a C18 column (ThermoFischer Scientific, Waltham, MA, USA).

### 2.7. Hemagglutination Assay

Recombinant HA_18–528_ and HA-PR8 were diluted with 1× phosphate buffer saline (PBS) to a concentration of 200 μg/mL. 50 μL of protein solution was added to a U-shaped cell culture plate and serially diluted two-fold along the plate; afterwards, 50 μL of 0.5% turkey red blood cells (RBC) in PBS was added to each well. PBS alone and recombinant TurboRFP were used as negative controls. After 30 min of incubation at room temperature, agglutination was assessed.

### 2.8. Mice Immunization and Challenge Infection with Influenza Virus A H1N1

Animal studies were approved by the Ethics Commission (25/24.03.2020) and endorsed by IDSA Bucharest, Romania, no. 522/12.06.2020. Methods for animal handling and care were carried out in accordance with relevant guidelines and regulations within the authorized animal facility of the “Cantacuzino” National Medico-Military Institute for Research and Development.

Three groups of five female BALB/c mice (Charles River), age 6–8 weeks, were inoculated intramuscularly with a 3 μg/50 μL dose of newly synthesized HA_18-528_, HA-PR8, and diluent (5% glucose, 0.1 × PBS). HA_18–528_ was treated with Pierce™ High Capacity Endotoxin Removal Resin (ThermoFischer Scientific, Waltham, MA, USA), and all samples were tested for bacterial lipopolysaccharide contamination with the LAL Chromogenic assay (HycultBiotech, Uden, The Netherlands) prior to inoculation. The procedure was repeated on day 21 for a second inoculation. Sera were collected on days −1 and 42. On day 56, mice were exposed to an 18 × LD_50_ dose of mouse-adapted influenza virus A/Puerto Rico/8/1934 H1N1 delivered by nebulization in individual cages. Weight variation was recorded daily. Survival data was analyzed using a log-rank test in R 4.2. After 9 days, all animals were euthanized.

### 2.9. Humoral Immune Response Evaluation

Specific antibodies in mouse serum were detected and quantified by end-point titration using an enzyme-linked immunosorbent assay (ELISA). Briefly, 384-well micro-titer plates (Nunc) were coated with 0.5 μg/mL of HA_18–528_ or HA-PR8 diluted in PBS and incubated overnight at 4 °C. The unreacted sites were blocked with 1% bovine serum albumin (BSA) in PBS for 2 h at room temperature (RT). Plates were washed three times with 0.05% Tween-80 in PBS, followed by the addition of serum samples. Sera obtained from sham mice served as a negative control. Individual serum samples were serially diluted from 1:200 to 1:6400 in 1% BSA and 0.05% Tween-80 in PBS. One pool of sera from antigen immunization at 42 days was also added in serial dilution from 1:50 to 1:102,400 to normalize results. After washing three times, goat anti-mouse IgG, IgG1, and IgG2a—HRP conjugated antibodies (SouthernBiotech, Birmingham, AL, USA), respectively, were added at appropriate dilutions to different assay plates. Following 1 h of incubation at RT and three washes, TMB (TetraMethylBenzidine) peroxidase substrate (- ThermoFischer Scientific, Waltham, MA, USA) was added to the wells. After color development, the reaction was stopped by the addition of 2N H_2_SO_4_, and the absorbance was measured at 450 nm (ThermoScientific Multiskan FC instrument). Analysis was performed in Rstudio version 1.2.5033 using a 4-parameter logistic curve with nplr (n-parameter logistic regression) and calibFit R packages and was represented as box plots using the ggplot2 package [29]. Results were expressed as endpoint titers, defined as the reciprocal sample dilution that would result in three times the baseline + standard error, as derived from the internal standard curve by multiplication. Each mouse serum sample was tested separately, in triplicate.

### 2.10. Hemagglutination Inhibition Assay

Allantoic fluid from embryonated chicken eggs that were infected with mouse-adapted influenza virus A/Puerto Rico/8/1934 H1N1 was diluted to 4 HA units, dispensed in a microplate, and incubated for 30 min with binary dilutions of mouse sera. Afterwards, 0.5% turkey RBC was added, and hemagglutination was recorded after an incubation of 30 min.

## 3. Results

### 3.1. Hemagglutinin Extracellular Domain Was Expressed in Bacterial Host

**Figure 2:**
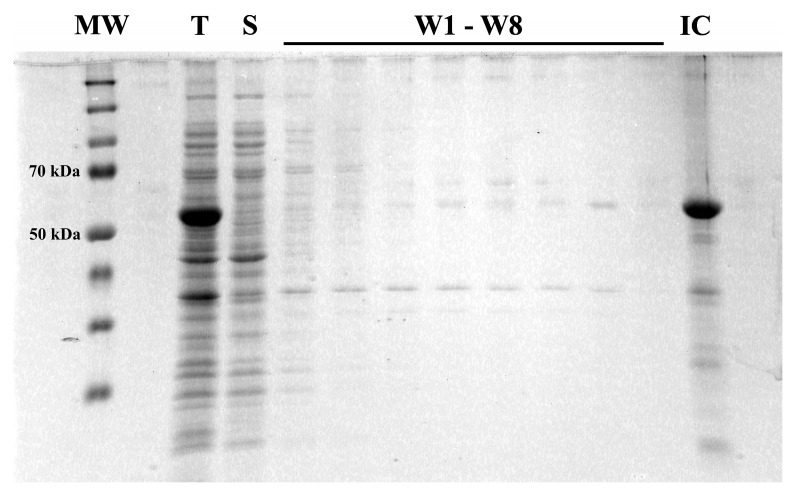
Bacterial expression of the hemagglutinin ectodomain, HA_18–528_. SDS-PAGE evaluation of relative amounts of expressed protein during the processing steps. MW—Spectra™ Multicolor Broad Range Protein Ladder; T—total lysate; S—soluble fraction; W1–W8—sequential washes; IC—inclusion bodies.

A new plasmid vector was constructed using a pET24a-(+) harboring the coding sequence for His-tagged full-length hemagglutinin (without signal peptide) of influenza virus A/Puerto Rico/8/1934 H1N1 as a template. Heterostagger-PCR and LIC were employed to excise the region corresponding to the transmembrane and cytoplasmic domains. The resulting vector was transformed into a bacterial host that, subsequently, was subjected to inducible expression of the hemagglutinin extracellular domain, HA_18–528_. Bacterial expression resulted in the accumulation of proteins exclusively in insoluble fractions in the form of inclusion bodies (Figure 2). Repeated dispersion in lysis buffer and ultrasonication improved the purity of the target protein from 24% in total lysate to approximately 60% in washed inclusion bodies (as estimated from SDS-PAGE).

### 3.2. Rapid Dilution Protein Refolding Optimization Using DSF

**Figure 3:**
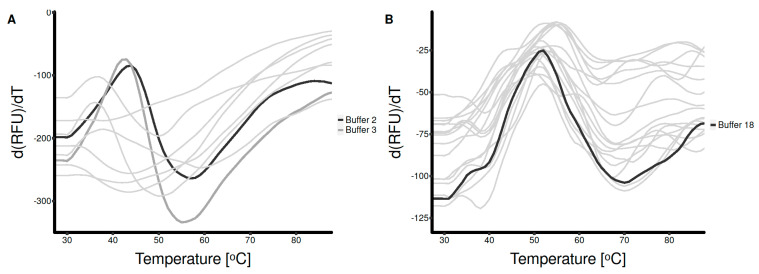
Screening for best refolding and dialysis conditions for HA_18–528_ using differential scanning fluorimetry. (**A**) The first screening round identified Buffers 2 and 3 (black and dark gray curves) as suitable for promoting refolding. (**B**)—Third screening round showing selected dialysis condition Buffer 18 (black curve). Thin, light gray curves in both figures represent data for other tested conditions.

Protein from inclusion bodies solubilized with guanidine hydrochloride was diluted in buffer solutions containing various additives that are known to promote protein refolding. The optimal buffer composition was decided using a two-step DSF screening process. The first screening round identified two suitable refolding buffer candidates (Figure 3A) out of nine tested conditions, with T_m_ of 44 °C and 43 °C, respectively. These were used as starting points for the second screening round, where the influence of polyethylene glycol (PEG 3350), NaCl, L-arginine, and GSH/GSSG was further investigated. All tested conditions in the second screening round performed comparably in terms of both T_m_ and amplitude of change in fluorescence (Supplementary Figure S4). Nevertheless, based on overall performance at each timepoint, Buffer 3–10 (50 mM Tris-HCl, 0.8 M L-arginine, GSH/GSSG 5:1, 0.06% PEG 3350, 20 mM NaCl) was selected as the best refolding buffer.

**Figure 4:**
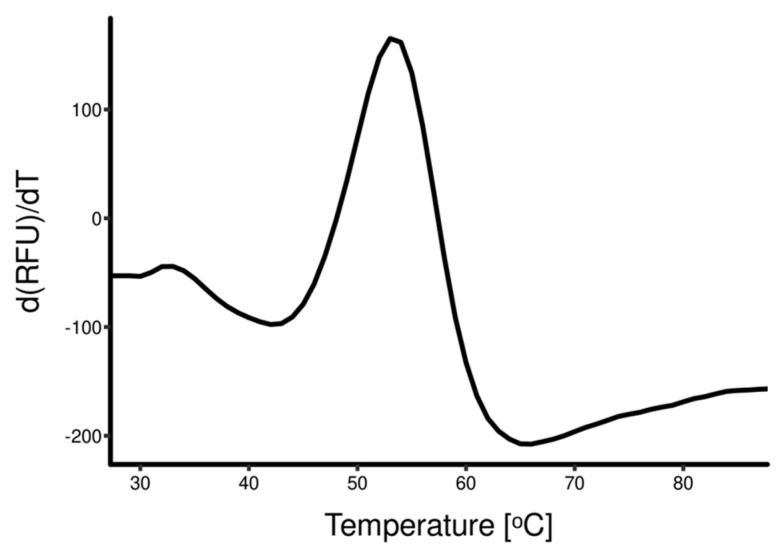
DSF profile of protein solution after refolding, dialysis, and purification. Dialysis was performed using 50 mM Tris-HCl and 100 mM NaCl at pH 8.5 overnight, and purification and concentration were performed on a Ni-NTA column.

To further improve the stability of the refolded protein, a third DSF screening round was performed to establish the composition of an optimal dialysis buffer. Refolded HA_18–528_ was diluted in 18 buffers that differ in pH (pH 6 to pH 8.5) and NaCl concentration (0–100 mM). The optimal composition of dialysis buffer, which consisted of 50 mM Tris-HCl and 100mM NaCl, pH 8.5, increased the T_m_ of HA_18–528_ to 52 °C (Figure 3B). Subsequent dialysis and purification improved the DSF profile of the protein solution and, moreover, slightly increased protein stability, T_m_ = 53 °C (Figure 4).

### 3.3. Refolded Hemagglutinin Consists of a Mix of Soluble Monomers and Oligomers with Biological Activity

After dialysis, protein solution was concentrated and purified simultaneously on IMAC (Immobilized Metal Chelate Affinity Chromatography) (Supplementary Figure S5), and, afterwards, imidazole was removed using a gel filtration column. Total HA_18–528_ protein yield, after purification and imidazole removal (Supplementary Figure S6), was approximately 25–40 mg per liter. Identity was confirmed by LC-MALDI MS analysis of tryptic peptides. Protein sequence coverage was 56.3% as computed with ProteinScape 4.0 using the theoretical digest method, with identified peptides covering both protein termini (Supplementary Figure S7).

**Figure 5:**
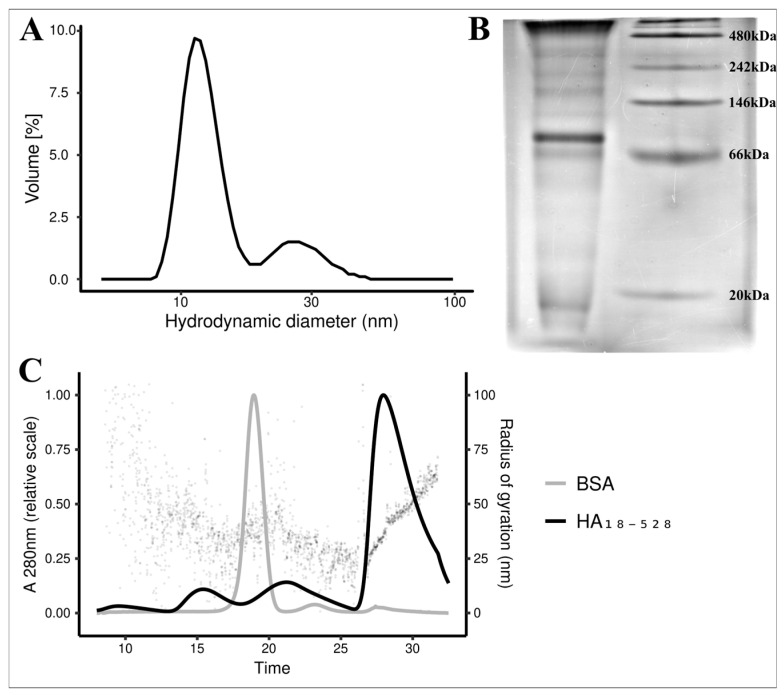
Supramolecular HA_18–528_ protein assembly. (**A**)—DLS particle size analysis of a 0.5 mg/mL HA_18–528_ protein solution. (**B**)—BN-PAGE of 30 μg HA_18–528_ on a 10—20% gradient Bis-Tris gel; Lane 1—HA_18–528_; Lane 2—NativeMark™ Unstained Protein Standard. (**C**)—FFF-MALS analysis of HA_18–528_; black line—HA_18–528_; gray line—BSA control; dots—radius of gyration calculated for HA_18–528_; separation under a 4 mL/min constant crossflow until t = 26 min, reduced to 0 mL/min (linear gradient, 1 min) at t = 27 min, and held constant until t = 32.5 min.

Protein solution consists of both soluble monomers and various oligomer forms, as demonstrated by particle size analysis and native gel electrophoresis (Figure 5). DLS data reveals two distinct populations characterized by hydrodynamic radii of roughly 11 nm and 27 nm. However, BN-PAGE shows one main population (probably the monomer form), accompanied by multiple other supramolecular states, some of which exceeded the resolving power of the 10–20% gradient Bis-Tris gel. In line with these results, FFF-MALS analysis showed that around 75% of the protein (by UV absorption) was found in higher oligomeric states, only eluting at low crossflow (Figure 5C). The high-MW oligomers had a calculated radius of gyration of 39.5 nm. No significant quantity of refolded protein was found in aggregates exceeding 100 nm in radius of gyration (Supplementary Figure S8).

**Figure 6:**
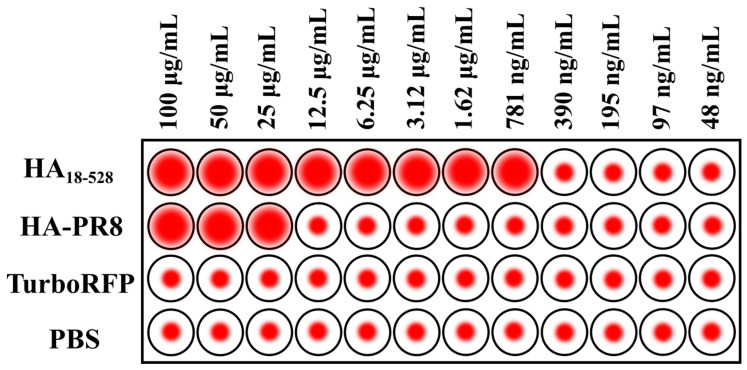
Agglutination of turkey RBC by HA_18–528_. Serial dilutions of the sample were mixed with washed RBC, and hemagglutination was evaluated after a 30 min incubation at RT. HA-PR8 was used at similar dilutions for comparison. PBS and TurboRFP served as negative controls.

Functional analysis of the refolded protein was performed by testing specific binding to sialic acid residues on turkey red blood cells. Hemagglutinin-mediated agglutination of RBC occurred at concentrations of 781 ng/mL. In contrast, HA-PR8 only promoted agglutination at higher concentrations (Figure 6).

### 3.4. Immunization of Mice with HA_18–528_ Generates Neutralizing Antibodies Coupled with a Balanced Th1/Th2 Immune Response and Protects against Viral Challenge

To test the newly synthesized protein’s ability to induce a protective immune response, BALB/c mice were inoculated with two doses, three weeks apart, of 3 μg of refolded and purified HA_18–528_. Two additional mice groups were similarly inoculated with HA-PR8 and diluent. 

Sera collected at days −1 and 42 post-immunization were tested for antigen-specific IgG antibodies and for cross-reactivity with other immunogens. IgG1 and IgG2a isotypes were also tested to assess the Th1/Th2 immune response bias (Supplementary Figure S9).

**Figure 7:**
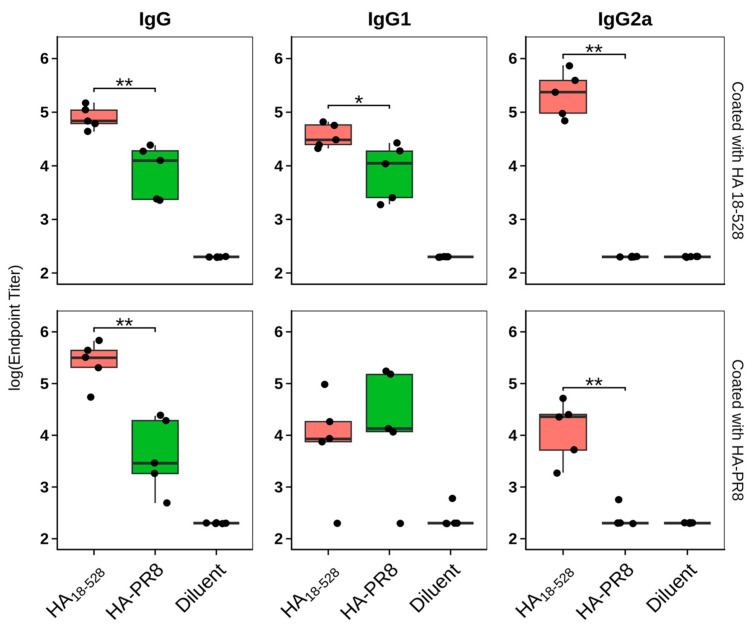
Antigen-specific antibody titers on day 42 of mice immunized with two doses of HA_18–528_, HA-PR8, or diluent. All samples were measured on both plates coated with HA_18–528_ and plates coated with HA-PR8. Black dots represent individual samples. Statistical analysis was performed using the Wilcoxon unpaired test (* *p* < 0.05, ** *p* < 0.01).

On day 42, samples from mice immunized with either antigen showed the presence of antigen-specific IgG antibodies, with higher titers in samples from mice that received HA_18–528_ (Figure 7). Also, immunization with both proteins induced IgG1 antigen-specific antibodies at comparable levels, while, surprisingly, only HA_18–528_ was able to elicit measurable levels of the IgG2a isotype (Figure 7). Sera cross reactivity was observed towards both tested antigens, HA_18–528_ and HA-PR8; similar patterns were observed when assaying IgG and IgG1, irrespective of the protein used for the coating of ELISA plates.

Day 42 sera were checked for neutralization activity against influenza virus A/Puerto Rico/8/1934 H1N1 using the hemagglutination inhibition assay. Results (Table 2) show that sera from mice immunized with HA_18–528_ (except for one sample) were able to block attachment of virus to sialic acid residues of turkey RBC, whereas sera from mice immunized with HA-PR8 or diluent did not.

**Figure 8:**
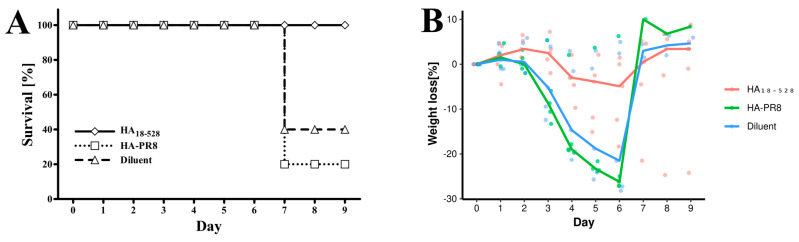
Survival and weight loss of immunized mice when challenged with the mouse-adapted influenza virus A/Puerto Rico/8/1934 H1N1. Mice were split into three groups that received a two-dose immunization (3 μg each) with HA_18–528_, HA-PR8, or diluent. (**A**)—survival curves, log-rank *p* = 0.05 HA_18–528_ group vs. diluent controls, *p* = 0.01 HA_18–528_ group vs. HA-PR8 group; (**B**)—weight loss calculated as % from day 0. Dots represent each individual measurements.

On day 56, all mice received an 18 × LD_50_ dose of the mouse-adapted influenza virus A/Puerto Rico/8/1934 H1N1. All mice immunized with HA_18–528_ survived the viral challenge, while only one mouse (out of five) survived to day 9 post-infection in the group that received HA-PR8, and two mice survived in the diluent control group (Figure 8A). Results were significant when comparing the HA_18–528_ group with either of the control groups (*p* = 0.05 HA_18–528_ vs. diluent, *p* = 0.01 HA_18–528_ vs. HA-PR8, log-rank test). Maximum weight loss was recorded on days 5–6 post-infection and surviving mice were subsequently recovered by day 9 (Figure 8B).

## 4. Discussion

Mainstream approaches to influenza vaccination still rely on the use of whole virus vaccines in the form of either live attenuated or inactivated viruses. Yet, despite good immunogenicity, high reactogenicity, and the inconveniences associated with the multiplication of live viruses, there is a demand for modern alternatives, such as subunit vaccines. These consist of purified viral surface proteins, the most common choice being hemagglutinin due to presence of neutralizing epitopes on its surface [30].

Although *E. coli* is widely considered the workhorse for recombinant protein production, protein expression in these hosts is seldom used in vaccine manufacturing, especially due to the poor solubility and improper folding of the obtained proteins. There are multiple studies that report successfully obtaining influenza A hemagglutinins by heterologous expression in bacteria [31,32,33,34], but the general agreement is that the methods used are specific for each hemagglutinin sequence. It has been reported that the correct conformation of bacterial HAs is highly dependent on the HA fragment chosen for expression and the refolding method. As most neutralizing epitopes are conformational, the native structure of hemagglutinin is desirable, and of special interest is the globular domain, which harbors most of these epitopes. One difficulty in the properly folded production of bacterial HA is the presence of large hydrophobic regions that are recognized as misfolded and undergo degradation. On the other hand, soluble HA proteins forming stable oligomeric structures have a significant influence on the quality of the immune response. Shorter bacterially expressed polypeptides have a better chance of folding to their native conformation, so most previous attempts made use of HA1 or, less frequently, HA2 regions when using bacterial hosts [35]. Larger domains, such as the ectodomain, have the potential to be more immunogenic and to better simulate native structure [35].

In this case, proper folding is challenging. A large variety of folding conditions need to be tested to find the optimal ones. A high-throughput evaluation method such as differential scanning fluorimetry could facilitate such screening.

In this work, we aimed to obtain, through bacterial recombinant protein expression, a soluble influenza A hemagglutinin fragment that would both present native-like folding and elicit a protective immune response in vivo. To this extent, we modified the sequence of influenza A/Puerto Rico/8/1934 H1N1 hemagglutinin by removing the transmembrane and the cytoplasmic regions, thus obtaining the ectodomain. In doing so, we sought to improve solubility, as the highly hydrophobic transmembrane region could impede proper protein folding and promote protein aggregation. Throughout the study, the complete HA0 protein sequence (HA-PR8), purified under denaturing conditions by preparative SDS-PAGE, was used for comparison in functional assays.

Despite truncation of both transmembrane and cytoplasmic domains, HA_18–528_ protein expression in bacteria still resulted in the accumulation of protein in inclusion bodies under the conditions tested. Studies have shown that proteins in inclusion bodies can retain a good deal of their secondary structure and even some native-like folding [36]. Also, as the composition of inclusion bodies is generally dominated by the overexpressed protein of interest, the number of purification steps required is usually lower. Therefore, instead of optimizing protein expression conditions for enhanced solubility, we chose to attempt refolding the abundant inclusion body-trapped protein. We hypothesized that, even though deletion of the transmembrane region did not directly result in soluble proteins, the absence of this highly hydrophobic domain could prevent aggregate formation and allow for easier refolding.

Inclusion bodies underwent solubilization with guanidinium hydrochloride, and reversal to a native-like structure was attempted by means of rapid dilution in different refolding cocktail solutions. Our approach was to screen for the optimal composition of refolding solutions using differential scanning fluorimetry (DSF), also called thermal shift assay or thermofluor assay. DSF is a high-throughput method that uses solvatochromic fluorescent dyes to discern folded from unfolded states of proteins by evaluating the variation in the amount of exposed hydrophobic regions as a function of temperature. As temperature increases, proteins unfold, and hydrophobic regions that are usually buried are exposed and may interact with hydrophobic fluorescent dyes, such as SYPRO Orange. Upon binding, the fluorescent dyes exhibit solvatochromism, and the fluorescent signal is used to plot the melting temperature of proteins (T_m_) [37]. Two sequential screening rounds were performed to find the optimal refolding cocktail composition. The maximum melting temperature that was achieved was T_m_ = 44 °C. From all the conditions tested, L-arginine and oxidized/reduced glutathione were the main factors that contributed to the folding of HA_18–528_. These findings confirm other studies that have shown that a redox environment is crucial for folding, as many proteins possess disulfide bridges that stabilize their correct conformation. Also, L-arginine is known to suppress protein aggregation and enhance refolding [38]. Nevertheless, chemical additives and their optimal concentrations and ratios are hard to predict. The use of DSF screening provided a high-throughput investigation that allowed us to choose an optimal refolding buffer composition. A further DSF screening was performed to pick the optimal stabilization buffer to be used for improved dialysis conditions when removing refolding agents [27]. This sequential screening improved the stability of the folded protein as T_m_ increased to 53 °C.

The native form of influenza hemagglutinin is a homotrimer resulting from the non-covalent association of HA0 monomers stabilized by disulfide bond formation and cleaved later on into two disulfide-linked components—HA1 and HA2 [39]. Regarding trimerization and folding, each domain seems to have its own role. Khurana et al. claimed that HA2 of H1N1 A/California/07/2009 is not needed for trimer formation and, moreover, recombinant HA1 (1–330), but not HA (1–480), formed functional oligomers [40]. Yet, part of HA2 contributes to the stem region of hemagglutinin, which is of special interest as it possesses highly conserved epitopes that sparked new hope for an “universal” influenza vaccine [41]. Our results show that the HA ectodomain of H1N1 expressed in *E. coli* hosts can form functional oligomers. While the difference from Khurana et al. might stem from sequence dissimilarity, it might also be due to the choice of refolding conditions, considering that, in our case, the DSF screening allowed for a better selection of a refolding buffer.

The functionality of each HA domain is very different. HA1 is involved in cell binding, and HA2 is involved in cell penetration by membrane fusion following endosomal pH change. Hemagglutinin constructs that include HA2 can be in either a pre-fusion conformation or a post-fusion conformation. While the pre-fusion conformation is the native one and is understood to be the most relevant to vaccine design, the post-fusion conformation, transformed for membrane insertion, is highly unstable. This complex structure makes the optimization of protein refolding very difficult.

In this study, we hypothesized that a properly folded recombinant HA ectodomain could assemble into functional oligomers. In this regard, we tested our folded protein’s ability to induce hemagglutination of turkey red blood cells. Refolded HA_18–528_ induced hemagglutination starting at 781 ng/mL, while for HA-PR8, hemagglutination only occurred at a concentration at least 32 times higher. This result proves both the native-like function of HA_18–528_ and the presence of oligomeric states that are able to induce hemaglutination. Further confirmation was provided by DLS particle analysis and PAGE separation in non-denaturing conditions that revealed a mix of monomeric and oligomeric states within the protein solution.

We further tested whether vaccination of BALB/c mice with HA_18–528_ could elicit a protective immune response. HA-PR8 was used as a denatured protein control. Prime-boost i.m. immunization with a low dose (3 μg) of refolded and purified HA_18–528_ proved protective in an experimental infection with the live influenza virus with respect to both survival and weight loss. In contrast, severe weight loss (down to the cut-off for euthanasia) was observed in the majority of mice from groups that received sham vaccination or HA-PR8 (Figure 8B).

While at 42 days post-immunization, both HA_18–528_ and HA-PR8 led to the induction of relatively high titers of HA-specific total IgG and IgG1 (significantly higher for HA_18–528_), IgG2a was only detectable in sera from mice immunized with HA_18–528_. Additionally, 42-day sera from the HA_18–528_ immunized group displayed neutralizing activity when tested in a hemagglutination inhibition assay, whereas sera from other groups did not. Usually, BALB/c mice respond to influenza vaccines with a Th2-type immune response that is predominantly associated with IgG1 antibodies, while sera of mice that survive viral infection have mostly an IgG2a isotype that is associated with a Th1-type immune response. Also, IgG2a antibodies can activate the complement system, stimulate antibody-dependent cell-mediated cytotoxicity (ADCC), and are associated with higher vaccination efficacy and superior viral clearance [42,43]. Therefore, we speculate that in our immunization scheme, due to its native-like conformation and the tendency to form oligomers, HA_18–528_ was able to also display conformational epitopes, as compared to HA-PR8, which prompted a balanced Th1/Th2 response and stimulated the production of antibodies with neutralizing activity.

The aim of this paper is to show how guided refolding technology can be used to accelerate the generation of complex vaccine candidates. This approach can have a substantial impact on emergency vaccination. Does it apply to modern influenza hemagglutinin sequence-based vaccines? Nowadays, based on AI protein folding software, there has been a remarkable development. AlphaFold (based on Google DeepMind), or the competitor from Meta AI, succeeds in predicting the shape of hundreds of millions of proteins. We believe that complementing this with instrumental technologies such as the one shown in this paper could have a high impact on protein-based vaccines. 

## 5. Conclusions

The ectodomain of influenza H1N1 A/Puerto Rico/8/1934 hemagglutinin (HA_18–528_) was cloned into an *E. coli* host and was abundantly expressed in the form of inclusion bodies. Protein was solubilized with a chemical denaturant and subjected to differential scanning fluorimetry-guided refolding. Refolded protein was found to consist of a mix of monomer and oligomer forms and was able to induce RBC hemagglutination. Two doses of immunization with 3 μg of HA_18–528_ granted protection to mouse-adapted influenza virus A/Puerto Rico/8/1934 H1N1, while no significant difference in disease evolution was observed in mice vaccinated with a denatured HA-PR8 protein as compared to sham-vaccinated Sera analysis showed that HA_18–528_ immunized mice had a balanced Th1/Th2 immune response, coupled with the generation of antibodies with neutralizing activity.

## Figures and Tables

**Table 1:** ProtParam computed parameters for hemagglutinin-derived polypeptides.

	Number of Amino Acids	Molecular Weight [kDa]	pI
HA-PR8	556	62.6	7.26
HA_18–528_	519	58.7	7.02

**Table 2:** Hemagglutination inhibition antibody titers of day 42 sera from mice that received two doses of HA_18–528_, HA-PR8, and diluent.

	HA_18–528_					HA-PR8					Diluent				
Mouse ID	1	2	3	4	5	1	2	3	4	5	1	2	3	4	5
HI Titer	1/40	1/40	-	1/160	1/80	-	-	-	-	-	-	-	-	-	-

## Data Availability

The data presented in this study are available on request from the corresponding author.

## Supplementary Materials

The following supporting information can be downloaded at: https://www.mdpi.com/article/10.3390/vaccines11101520/s1, Figure S1: Amino acid sequence of HA-PR8 and HA_18–528_; Supplementary Figure S2: Analytical SDS-PAGE of HA-PR8; Supplementary Figure S3: DSF analysis of HA-PR8; Table S1: Base buffers in the first round of refolding optimization screening; Table S2: Selected buffers in the second round of refolding optimization screening; Table S3: Buffers included in dialysis DSF screening; Figure S4: Screening for optimal refolding and subsequent dialysis conditions for HA_18–528_ using DSF. Second screening round; Figure S5: IMAC chromatogram representing purification of HA_18–528_ using a HisTrap FF column (5 mL); Figure S6: Refolding, dialysis, and purification of the hemagglutinin ectodomain of HA_18–528_; Figure S7: LC-MALDI MS analysis of tryptic peptides; Supplementary Figure S8: FFF-MALS analysis of HA_18–528_; Figure S9: Antigen-specific antibody titers at days 1 and 42 of mice immunized with two doses of HA_18–528_, HA-PR8, or diluent.
