# Supplementary material for: Generation of a DSF-Guided Refolded Bacterially Expressed Hemagglutinin Ectodomain of Influenza Virus A/Puerto Rico/8/1934 H1N1 as a Model for Influenza Vaccine Antigens
Source: Vaccines (Basel). 2023 Sep 24;11(10):1520. doi: 10.3390/vaccines11101520 (PMC10610769; doi:10.3390/vaccines11101520)
Supplement: Supplementary file 1 [file vaccines-11-01520-s001.zip › vaccines-2596358-supplementary.pdf]

Generation of a DSF-Guided Refolded Bacterially Expressed Hemagglutinin Ectodomain of Influenza Virus A/Puerto Rico/8/1934 H1N1 as a Model for Influenza Vaccine Antigens

Supplementary

>HA-PR8

MDTICIGYHANNSTDTVDTVLEKNVTVTHSVNLLEDSHNGKLCRLKGIAPLQLGKCNIAGWLLGNPECD
PLLPVRSWSYIVETPNSENGICYPGDFIDYEELREQLSSVSSFERFEIFPKESSWPNHNTNGVTAACSHEGKSS
FYRNLLWLTEKEGSYPKLKNSYVNKKGKEVLVLWGIHHPPNSKEQQNLYQNENAYVSVVTSNYNRRFTPEI
AERPKVRDQAGRMNYYWTLLKPGDTIIFEANGNLIAPMYAFALSRGFGSGIITSNASMHECNTKCQTPLGA
INSSLPYQNIHPVTIGECPKYVRSAKLRMVTGLRNTPSIQSRGLFGAIAGFIEGGWTGMIDGWYGYHHQNE
QGSGYAADQKSTQNAINGITNKVNTVIEKMNIQFTAVGKEFNKLEKRMENLNKKVDDGFLDIWTYNAEL
LVLLENERTLDFHDSNVKNLYEKVKSQLKNNAKEIGNGCFEFYHKCDNECMESVRNGTYDYPKYSEESKL
NREKVDGVKLESMGIYQILAIYSTVASSLVLLVSLGAISFWMCSNGSLQCRICI

>$HA_{18–528}$

MDTICIGYHANNSTDTVDTVLEKNVTVTHSVNLLEDSHNGKLCRLKGIAPLQLGKCNIAGWLLGNPECD
PLLPVRSWSYIVETPNSENGICYPGDFIDYEELREQLSSVSSFERFEIFPKESSWPNHNTNGVTAACSHEGKSS
FYRNLLWLTEKEGSYPKLKNSYVNKKGKEVLVLWGIHHPPNSKEQQNLYQNENAYVSVVTSNYNRRFTPEI
AERPKVRDQAGRMNYYWTLLKPGDTIIFEANGNLIAPMYAFALSRGFGSGIITSNASMHECNTKCQTPLGA
INSSLPYQNIHPVTIGECPKYVRSAKLRMVTGLRNTPSIQSRGLFGAIAGFIEGGWTGMIDGWYGYHHQNE
QGSGYAADQKSTQNAINGITNKVNTVIEKMNIQFTAVGKEFNKLEKRMENLNKKVDDGFLDIWTYNAEL
LVLLENERTLDFHDSNVKNLYEKVKSQLKNNAKEIGNGCFEFYHKCDNECMESVRNGTYDYPKYSEESKL
NREKVDGVKLESMGIYQHHHHHH

Supplementary Figure S1. Aminoacids sequence of HA-PR8 and $HA_{18–528}$

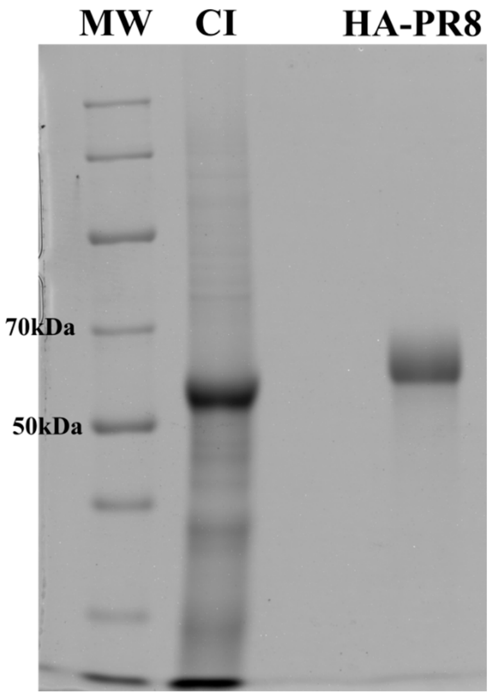

Supplementary Figure S2. Analytical SDS-PAGE of HA-PR8. MW - Spectra™ Multicolor Broad Range Protein Ladder. CI – HA-PR8 washed inclusion bodies. HA-PR8 – Purified HA-PR8.

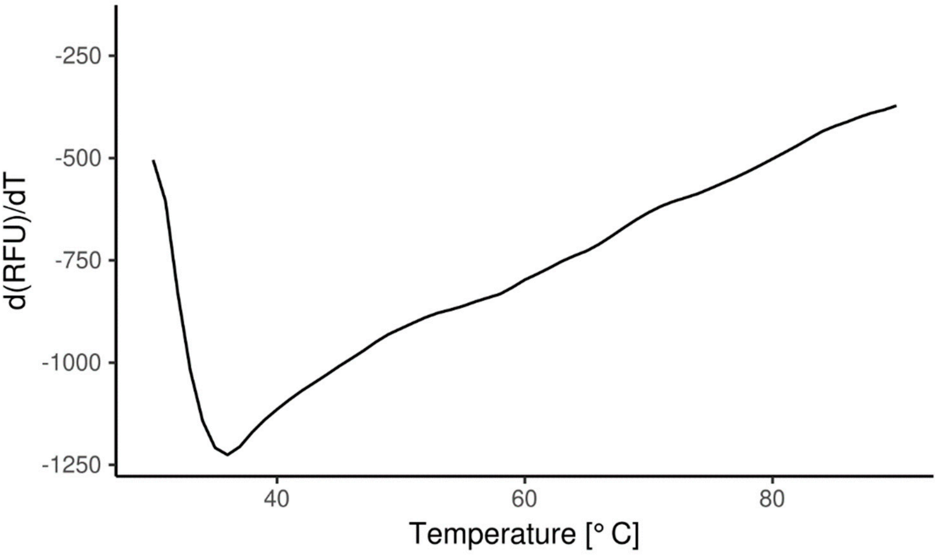

Supplementary Figure S3. DSF analysis of HA-PR8. 100ug of HA-PR8 in Tris-Glycine Buffer.

Base Buffer	Tris-HCl (mM)	NaCl (mM)	KCl (mM)	L-Arg (mM)	GdHCl (mM)	Redox conditions
**1**	50	20	0.8	0	0	5 mM DTT
**2**	50	20	0.8	400	0	2 mM GSH<br>0.2 mM GSSG
**3**	50	20	0.8	800	0	2 mM GSH<br>0.4 mM GSSG
**4**	50	20	0.8	0	500	2 mM GSH<br>0.2 mM GSSG
**5**	50	20	0.8	400	500	2 mM GSH<br>0.4 mM GSSG
**6**	50	20	0.8	800	500	5 mM DTT
**7**	50	20	0.8	0	1000	2 mM GSH<br>0.4 mM GSSG
**8**	50	20	0.8	400	1000	5 mM DTT
**9**	50	20	0.8	800	1000	2 mM GSH<br>0.2 mM GSSG

Supplementary Table S1. Base buffers in the first round refolding optimization screening

Refolding Buffer	Base Buffer	GSH: GSSG ratio	PEG330 (%)	NaCl (mM)
**2-1**	2	10:1	0	20
**2-2**	2	10:1	0	50
**2-3**	2	10:1	0	150
**2-4**	2	10:1	0.06	20
**2-5**	2	10:1	0.06	50
**2-6**	2	10:1	0.06	150
**2-7**	2	5:1	0	20
**2-8**	2	5:1	0	50
**2-9**	2	5:1	0	150
**2-10**	2	5:1	0.06	20
**2-11**	2	5:1	0.06	50
**2-12**	2	5:1	0.06	150
**3-1**	3	10:1	0	20
**3-2**	3	10:1	0	50
**3-3**	3	10:1	0	150
**3-4**	3	10:1	0.06	20
**3-5**	3	10:1	0.06	50
**3-6**	3	10:1	0.06	150
**3-7**	3	5:1	0	20
**3-8**	3	5:1	0	50
**3-9**	3	5:1	0	150
**3-10**	3	5:1	0.06	20
**3-11**	3	5:1	0.06	50
**3-12**	3	5:1	0.06	150

Supplementary Table S2. Selected buffers in the second round refolding optimization screening

Dialysis buffer	pH	Buffer	NaCl (mM)
**1**	6	50 mM Bis-Tris	0
**2**	6	50 mM Bis-Tris	50
**3**	6	50 mM Bis-Tris	100
**4**	6.5	50 mM Bis-Tris	0
**5**	6.5	50 mM Bis-Tris	50
**6**	6.5	50 mM Bis-Tris	100
**7**	7	50 mM HEPES	0
**8**	7	50 mM HEPES	50
**9**	7	50 mM HEPES	100
**10**	7.5	50 mM HEPES	0
**11**	7.5	50 mM HEPES	50
**12**	7.5	50 mM HEPES	100
**13**	8	50 mM Tris-HCl	0
**14**	8	50 mM Tris-HCl	50
**15**	8	50 mM Tris-HCl	100
**16**	8.5	50 mM Tris-HCl	0
**17**	8.5	50 mM Tris-HCl	50
**18**	8.5	50 mM Tris-HCl	100

Supplementary Table S3. Buffers included in dialysis DSF screening.

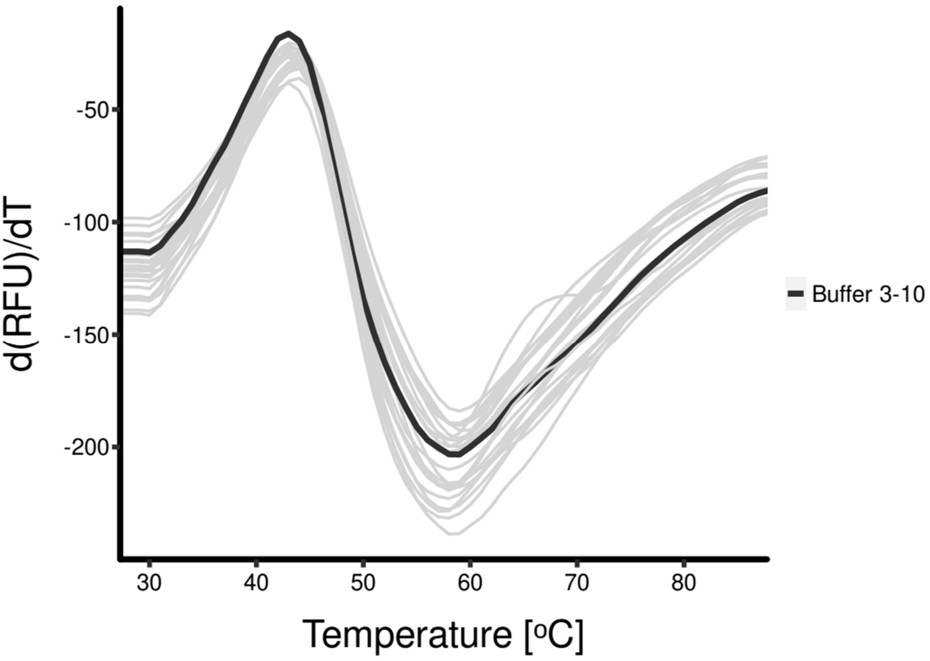

Supplementary Figure S4. Screening for optimal refolding and subsequent dialysis conditions for $HA_{18–528}$ using DSF. Second screening round. Tested conditions performed comparably. Buffer 3-10 was selected for further experiments.

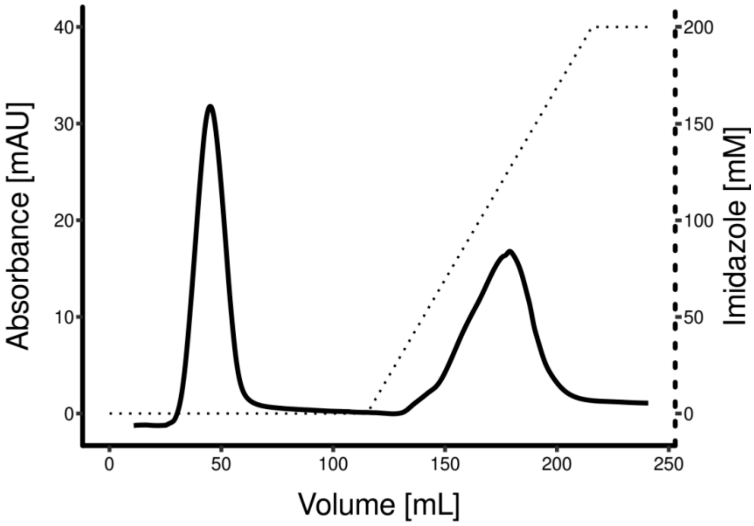

Supplementary Figure S5. IMAC chromatogram representing purification of $HA_{18–528}$ using a HisTrap FF column (5mL). Dotted line shows imidazole concentration and solid line shows absorbance at λ=280nm. First peak corresponds to flow-through, while the second peak corresponds to purified protein.

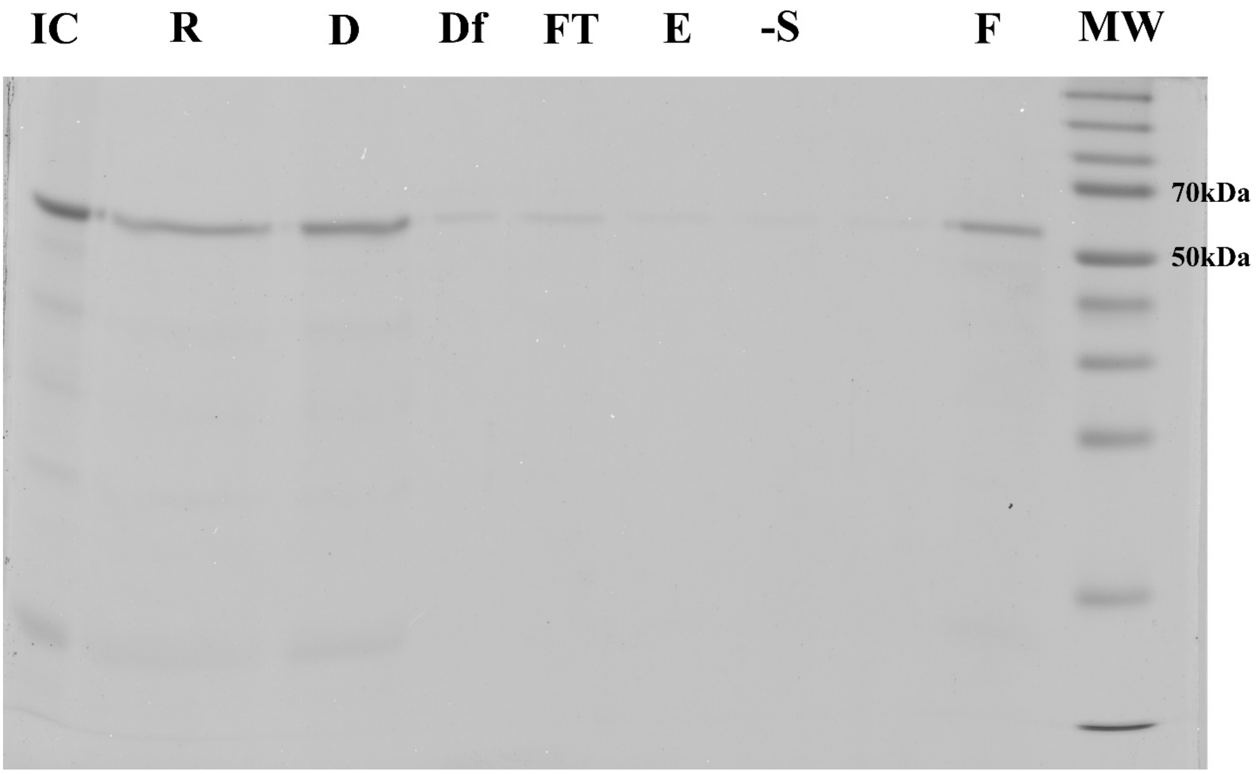

Supplementary Figure S6. Refolding, dialysis and purification hemagglutinin ectodomain, $HA_{18–528}$. SDS-PAGE evaluation of relative amounts of protein during the processing steps. IC – Inclusion bodies, R – refolded protein, D – dialyzed protein, Df – 0.22μm filtered dialyzed protein, FT – IMAC flow-through, E – IMAC elution, -S – desalted protein solution, F – final protein product, MW - Spectra™ Multicolor Broad Range Protein Ladder. Samples were loaded at theoretical equal amounts, except for F, which has ten times the theoretical amount.

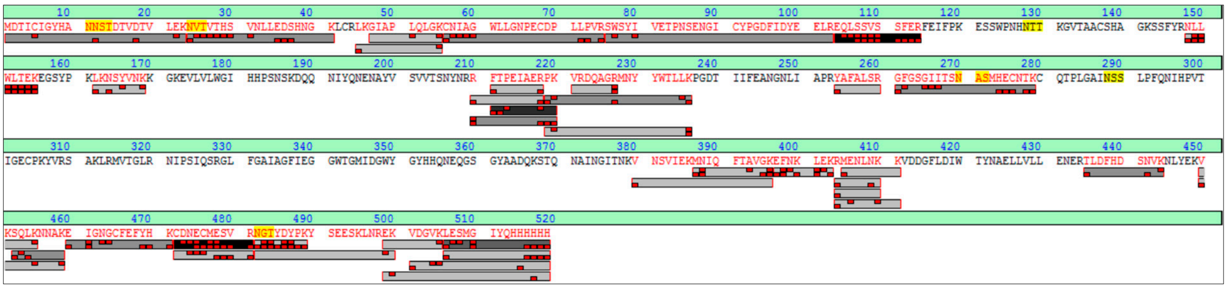

Supplementary Figure S7. LC-MALDI MS analysis of tryptic peptides. $HA_{18–528}$ sequence coverage as computed with ProteinScape 4.0 using the theoretical digest method.

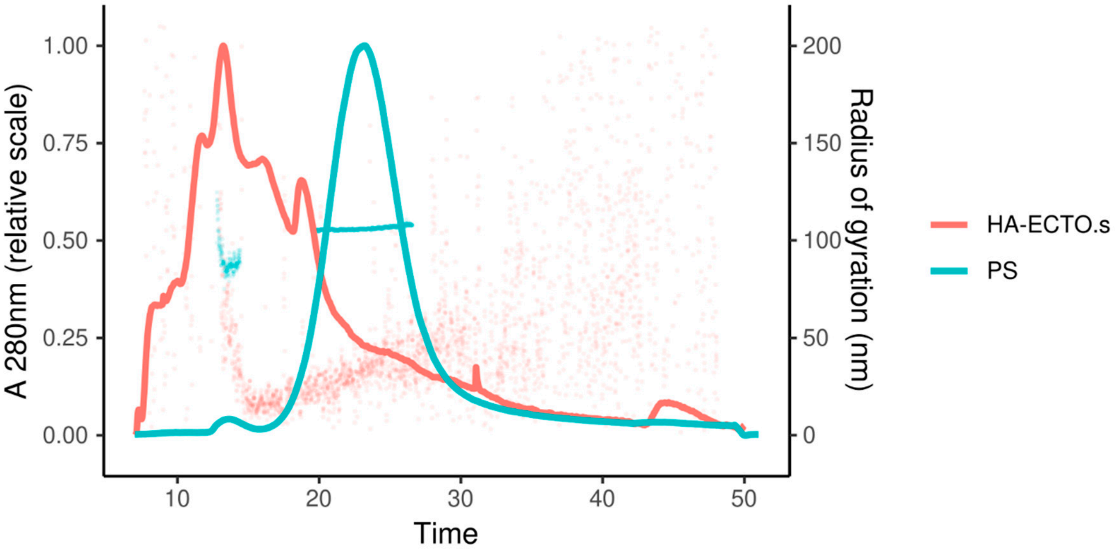

Supplementary Figure S8. FFF-MALS analysis of $HA_{18–528}$; red - $HA_{18–528}$; turquoise – 190nm diameter polystyrene beads control; dots – calculated radius of gyration; separation under an exponential crossflow gradient from 2.5mL to 0.05mL/min (t = 7min – t = 27min), held constant at 0.05mL/min until t = 42min and decreased to 0mL/min until t = 50min.

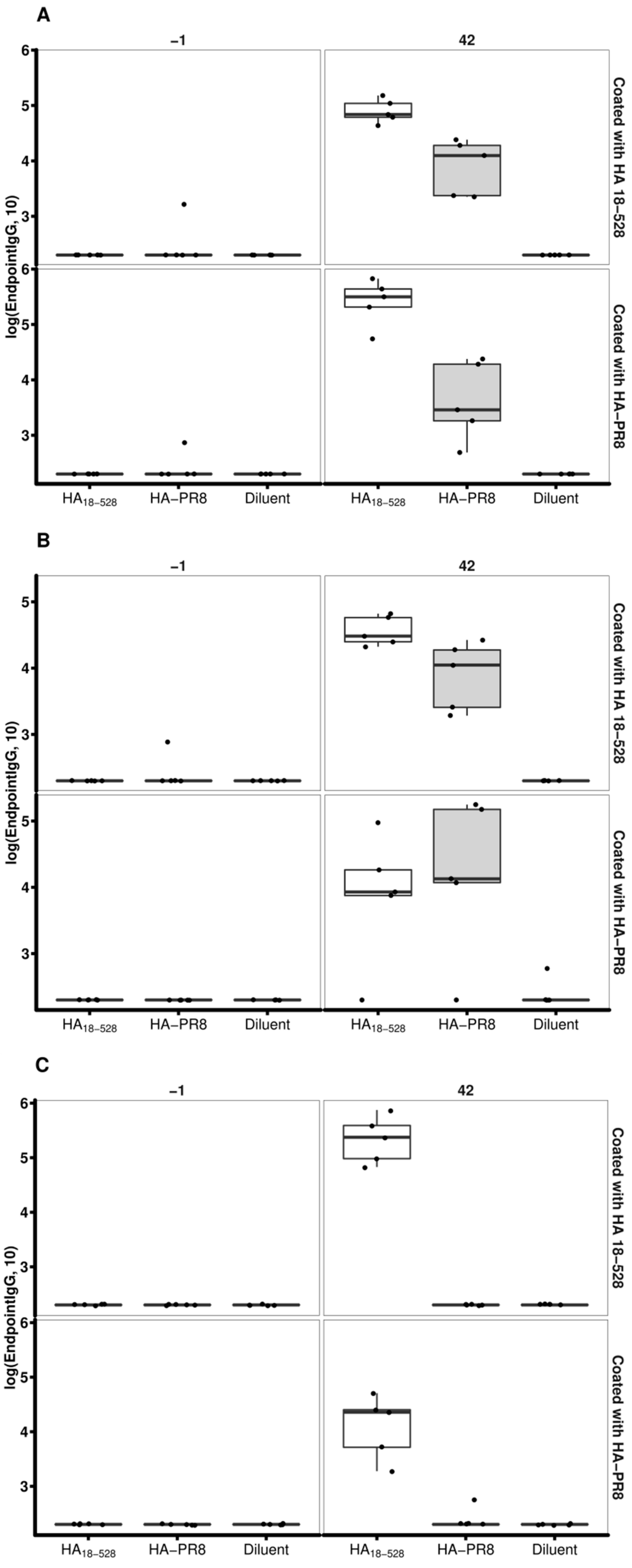

Supplementary Figure S9. Antigen specific antibodies titers at day -1 and day 42 of mice immunized with two doses of $HA_{18–528}$, HA-PR8 or diluent. All samples were measured on both plates coated with $HA_{18–528}$ and plates coated with HA-PR8. A – mouse IgG, B – mouse IgG1, C – mouse IgG2a.
